# Natural language processing: put your model where your mouth is

**DOI:** 10.15252/msb.20178077

**Published:** 2017-12-18

**Authors:** Rachel A Haggerty, Jeremy E Purvis

**Affiliations:** ^1^ Department of Genetics University of North Carolina at Chapel Hill Chapel Hill NC USA

**Keywords:** Computational Biology, Methods & Resources, Signal Transduction

## Abstract

Molecular mechanisms are often described using “word models”—phrases intended to capture the interactions in a biological process. In their recent work, Sorger and colleagues (Gyori *et al*, 2017) provide a framework for converting word models into computational structures that can be simulated and compared to experimental data. By codifying word‐based descriptions of molecular phenomena, scientific communities can better evaluate, compare, and share mechanistic insights.

Any model of biological mechanism—whether described in words, diagrams, or some other form—is only as good as the quality of its predictions. Models that consistently provide accurate predictions are likely to reflect at least some aspect of biological reality. Although the importance of predictive power is generally appreciated, however, it is not always obvious what the exact predictions of a particular model should be. The cell signaling literature abounds with phrases such as “MEK activates ERK”, “Rho inhibits Rac”, or “Wnt binds to Frizzled” that—though perhaps accepted to be true statements—do not always provide unambiguous, quantitative predictions about how the system will behave over time. This makes it difficult to compare word models to experimental data and, as a consequence, to evaluate whether the model is any good. Thus, a major challenge for the field of molecular systems biology is to determine whether language‐based descriptions of a molecular system can be directly encoded, simulated, and tested against experimental data. With this goal in sight, Gyori *et al* (2017) bridge natural language models and numerical simulations through a tool called INDRA (Integrated Network and Dynamical Reasoning Assembler) that interprets natural language into a series of statements that are assembled into machine‐executable equations. Through a series of field tests, INDRA shows that codifying our language‐based models places forcible weight on our words by turning them into immediately testable predictions (Fig 1).

**Figure 1:**
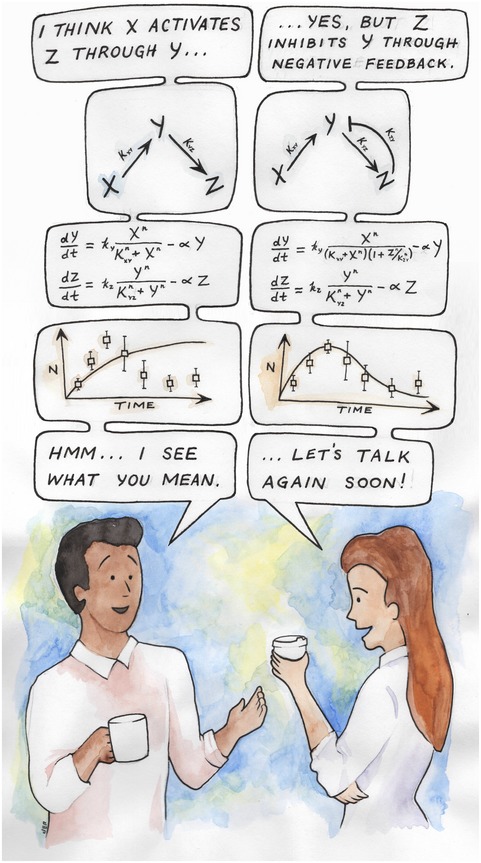
Translating word models into executable models allows direct, quantitative comparison of mechanistic hypotheses

Imagine the following task: Select a paper that describes how a particular molecular pathway works; use diagrams and text within the paper to build an executable model; and then compare the model's simulated output to the data reported in the same paper. In short, is the model presented in the paper consistent with the experimental results? The authors used INDRA to parse text describing the core p53 signaling network, a molecular circuit known to produce oscillations in response to DNA damage (Lahav *et al*, 2004). Simple declarative phrases such as “Active p53 activates Mdm2” and “Active Wip1 inactivates p53” were assembled into a series of ordinary differentiation equations and then simulated numerically. Perhaps unsurprisingly, these phrases alone were not sufficient to reproduce p53 oscillations. In order to capture the correct behavior, the authors had to add additional statements describing negative regulation from Mdm2 and Wip1—a known interaction that was omitted from the diagram in the paper for clarity. Apparently, when words are translated directly in executable models, it becomes clear that word models often lack critical details necessary to reproduce an observed behavior and that building accurate models may require additional input from experts.

A second test for INDRA involved comparing multiple mechanisms of drug resistance in melanomas (Larkin *et al*, 2014). Treatment of BRAF‐V600E tumors with small molecule inhibitors often leads to drug resistance and tumor regrowth, a behavior characterized by a transient drop in phosphorylated ERK (pERK) followed by a return in pERK levels. By explicitly formalizing different word models representing alternative mechanisms (Poulikakos *et al*, 2010; Lito *et al*, 2012; Yao *et al*, 2015), INDRA revealed that both pERK‐mediated feedback and BRAF dimerization were necessary for the observed pERK rebound and adaptive behavior. At minimum, they predicted, an accurate model of BRAF‐V600E drug resistance should contain both of these features. Thus, by evaluating and comparing multiple mechanistic hypotheses, INDRA can serve as a sort of computational arbiter that adjudicates competing ideas among scientists in a particular field.

Another benefit of using natural language processing for computational modeling is the potential to unify knowledge within a field. Different sub‐disciplines of molecular biology often have different linguistic norms for expressing molecular mechanisms. These differences include alternative uses of words, graphical representations, or mathematical descriptions. INDRA coverts ambiguities and assumptions into explicit mechanistic statements, employing a few simple rules that help to keep models from becoming overly complicated. In a third field test, INDRA reproduced a complex diagram of the Ras signaling pathway (the Ras Pathway 1.0) comprising findings from hundreds of papers and refined by an online discussion forum of Ras signaling experts (Stephen *et al*, 2014). Since INDRA starts with natural language processing, it can easily incorporate updates from many scientists by adding a list of phrases or sentences describing the molecular interactions. In this way, INDRA shows promise as a tool for codifying collective knowledge in a scientific field.

Yet the ability to parse natural language may also be one of INDRA's liabilities. Like all natural language processing programs, misspellings and grammatical errors can cause problems for interpretation, and the program may not be able to recognize all words in a word model (a process called “grounding”, in which each agent in a model is associated with a corresponding entry in a database of proteins, transcripts, or metabolites). In addition, more work is needed to extend INDRA to handle other types of biological entities such as short RNAs, lipids, and epigenetic marks. Compared to enzyme‐based signal transduction networks, these sub‐disciplines have relatively less well‐established databases and descriptive phrases for describing their molecular mechanisms.

It is unrealistic that a language processing tool like INDRA will fully automate the construction of accurate molecular models from the scientific literature. The authors are quick to note that, like any biological modeling project, building an accurate model usually requires multiple rounds of meaningful interaction between biological experts and the computational model. This was easily seen in the case of the p53 network, in which prior knowledge of, and intuition for, the system was required to reproduce the correct experimental behavior. As any experienced modeler will tell you, however, the need for expert curation should not be viewed as a limitation but rather as a benefit. Interaction with computational models builds quantitative and mechanistic insights for experimentalists beyond what can be acquired in a laboratory.

This study also reveals that building models from words may have the added benefit of making our conversational language more precise. For example, the casual phrase “ATM phosphorylates itself” was found to be inexact whereas the more precise phrase “active ATM phosphorylates another ATM molecule” (while perhaps awkward during a coffee‐break discussion) was able to produce the correct simulated behavior. Like all programming languages, INDRA does as it is told, not necessarily as it is intended. Thus, as INDRA works toward the long‐term goal of codifying a vast knowledgebase of biological mechanisms, we can build better models by choosing our words more carefully.
